# DNA Methyltransferase Controls Stem Cell Aging by Regulating BMI1 and EZH2 through MicroRNAs

**DOI:** 10.1371/journal.pone.0019503

**Published:** 2011-05-10

**Authors:** Ah-Young So, Ji-Won Jung, Seunghee Lee, Hyung-Sik Kim, Kyung-Sun Kang

**Affiliations:** 1 Adult Stem Cell Research Center, College of Veterinary Medicine, Seoul National University, Seoul, Republic of Korea; 2 Department of Veterinary Public Health, College of Veterinary Medicine, Seoul National University, Seoul, Republic of Korea; 3 Research Institute for Veterinary Science, College of Veterinary Medicine, Seoul National University, Seoul, Republic of Korea; 4 Division of Intractable Diseases, Center for Biomedical Sciences, Korea National Institute of Health, Chungbuk, Republic of Korea; Florida State University, United States of America

## Abstract

Epigenetic regulation of gene expression is well known mechanism that regulates cellular senescence of cancer cells. Here we show that inhibition of DNA methyltransferases (DNMTs) with 5-azacytidine (5-AzaC) or with specific small interfering RNA (siRNA) against DNMT1 and 3b induced the cellular senescence of human umbilical cord blood-derived multipotent stem cells (hUCB-MSCs) and increased p16^INK4A^ and p21^CIP1/WAF1^ expression. DNMT inhibition changed histone marks into the active forms and decreased the methylation of CpG islands in the p16^INK4A^ and p21^CIP1/WAF1^ promoter regions. Enrichment of EZH2, the key factor that methylates histone H3 lysine 9 and 27 residues, was decreased on the p16^INK4A^ and p21^CIP1/WAF1^ promoter regions. We found that DNMT inhibition decreased expression levels of Polycomb-group (PcG) proteins and increased expression of microRNAs (miRNAs), which target PcG proteins. Decreased CpG island methylation and increased levels of active histone marks at genomic regions encoding miRNAs were observed after 5-AzaC treatment. Taken together, DNMTs have a critical role in regulating the cellular senescence of hUCB-MSCs through controlling not only the DNA methylation status but also active/inactive histone marks at genomic regions of PcG-targeting miRNAs and p16^INK4A^ and p21^CIP1/WAF1^ promoter regions.

## Introduction

Cellular senescence is a significant mechanism for the maintenance of stem cell self-renewal and multipotency [1], [2]. Epigenetic regulatory mechanisms, such as acetylation and methylation of core histones, DNA methylation and microRNAs (miRNAs), have been reported to play pivotal roles in regulating cellular senescence [3]. We have previously shown that the inhibition of histone deacetylases (HDACs) induces cellular senescence of human multipotent stem cells (MSCs) by controlling the balance in the expression levels of polycomb group (PcG) and jumonji domain containing 3 (JMJD3) proteins [4].

DNA methyltransferase (DNMT) is an enzyme that catalyzes the transfer of a methyl group to DNA. DNA methylation is one of the regulatory mechanisms of gene expression by which transcriptional activity of DNA decreases and DNA stability increases. DNMT has multiple isoforms, including DNMT1, DNMT3A and DNMT3B, which have different roles. DNMT1 maintains methylation of DNA, while DNMT3A and DNMT3B make *de novo* DNA methylation. It is well known that DNMT over-expression induces aberrant hypermethylation, which contributes to silencing tumor suppressor genes in various cancer cells [5], [6], [7], [8], [9]. The promoter region of p16^INK4A^, a cyclin dependent kinase (CDK) inhibitor, is hypermethylated as a result of over-expression of DNMTs in many cancer cell lines [8], [10], [11]. The expression of p21^CIP1/WAF1^, another CDK inhibitor, is also regulated by DNA methylation [12]. Given that CDK inhibitors, p16^INK4A^ and p21^CIP1/WAF1^ are known key players in cellular senescence *in vitro* [13], [14], it was assumed that DNMTs might be involved in cellular senescence of stem cells; however, direct evidence whether DNMT involves in the regulation of stem cell aging has not been reported yet. Epigenetic regulatory machineries, such as DNA methylation, histone acetylation, deacetylation and histone methylation, are associated with and regulated by each other [15]. Although the primary role of DNMTs is to methylate DNA, DNMTs are also reported to modulate patterns of histone acetylation and methylation. Treatment with 5-azacytidine (5-AzaC), an inhibitor of DNMT analogous to cytidine, not only inhibits DNMT activity but also affects histone modification patterns, suggesting that DNMT may modulate core histone via both direct and indirect mechanisms [16]. PcG proteins are key factors that translate DNA methylation patterns into histone modifications. PcGs are comprised of two main PcG complexes, polycomb repressive complex (PRC) 1 and 2. PRC2 group proteins are involved in the initiation of gene silencing, whereas PRC1 stabilizes and maintains gene repression. It was reported that SUZ12, a PRC2 protein, recognizes and binds to methylated CpG in the genome. Binding of SUZ12 onto methylated CpG initiates recruitment of EZH2, another PRC2 protein possessing histone methyltransferase activity, to the site of DNA methlyation and induces methylation of histone H3 at the lysine 27 residue. BMI-1, a PRC1 protein, is recruited to the PRC2 complex and maintains transcriptional repression [17]. In addition, Jin et al. showed that DNMT3B plays an important role in controlling histone modification patterns by regulating PRC1 function [18]. Hernandez-Munoz I et al. confirmed that DNMT1 is necessary for proper assembly of the PRC body [19]. In contrast, histone modification patterns and other patterns of epigenetic modifiers influence the propensity of genes to become hypermethylated in cancer [18].

MiRNAs are non-coding RNAs and are smaller than 22 nucleotides. MiRNAs are epigenetic regulators of gene expression that degrade or inhibit translation of target mRNAs. In many studies, miRNAs have been reported to target oncogenes, tumor suppressors in cancer and differentiation markers, which should be silenced to keep stem cells from differentiating and instead self-renew [20], [21], [22], [23], [24], [25], [26]. MiRNAs can also regulate other epigenetic regulators such as DNMT, HDAC, high-mobility group AT-hook 2 (HMGA2) and PcG [27], [28], [29], [30], [31], [32], [33]. Although many studies have focused on the target of specific miRNAs, considering the importance of the biological roles of miRNAs, the intermediary mechanisms of miRNAs among the epigenetic regulatory factors should be explored. The possibility of epigenetic repression, mediated by DNA methylation and histone modification of tumor suppressor miRNAs in human cancer cells, has been reported [34]. Recently, there have been several reports of the epigenetic control of miRNA clusters [31], [35], [36]. Briefly, genomic DNA regions that encode tumor suppressor miRNAs are inactivated by aberrant hypermethylation in human breast cancer cell lines. After treatment with 5-aza-2′-deoxycytidine (5-Aza-dC), demethylation of the mir-9-1 genome and increases in mir-9-1 expression were observed [35]. In our previous study, we showed that inhibition of HDACs up-regulated miRNAs that target HMGA2. The data suggested that modification of histone patterns bound to genomic DNA regions of miRNAs may regulate miRNA expression by epigenetic control [31].

Taken together, the functions of the epigenetic regulatory factors DNMT, HDAC, PcG and miRNAs overlap and cross-regulate each other. Although the regulation of stem cell cellular senescence by DNMTs has been established, the biological role of DNMTs in stem cell self-renewal has yet to be elucidated. Here, we demonstrate a role of DNMT during cellular senescence of hUCB-MSCs, uncovering how epigenetic regulatory factors, such as HDAC, PcG and miRNAs, are involved in DNMT activity.

## Materials and Methods

### Isolation and culture of hMSCs

The UCB samples were obtained from the umbilical vein immediately after delivery, with the written informed consent of the mother approved by the Boramae Hospital Institutional Review Board (IRB) and the Seoul National University IRB(IRB No. 0603/001-002-07C1). The hUCB-MSCs were isolated and cultured as previously described [4], [37]. Briefly, The UCB samples were mixed with the Hetasep solution (StemCell Technologies, Vancouver, Canada) at a ratio of 5∶1, and then incubated at room temperature to deplete erythrocyte counts. The supernatant was carefully collected and mononuclear cells were obtained using Ficoll density-gradient centrifugation at 2,500 rpm for 20 min. The cells were washed twice in PBS. Cells were seeded at a density of 2×10^5^ to 2×10^6^ cells/cm^2^ on plates in growth media consisted of D-media (Formula No. 78-5470EF, Gibco BRL) containing EGM-2 SingleQuot and 10% fetal bovine serum (Gibco BRL). After 3 days, non-adherent cells were removed. For long term culture, cells were seeded at a density of 4×10^5^ cells/10 cm-plate and subcultured cells when they reach 80∼90% confluency.

### Senescence-associated beta-galactosidase (SA β-gal) staining

SA β-gal staining was carried out as described by Narita *et al.*, with some modifications [38]. The MSCs were seeded on 6-well plates at a density of 1×10^5^/well for late-passage cells and 5×10^4^/well for early-passage cells. Cells were incubated for 3 d until reaching the appropriate confluence. For siRNA or anti-miRNA treatment, cells were seeded at a density of 2×10^4^/ml, and siRNA or anti-miRNA was used to treat the cells at 50–60% confluence. The cells were washed twice with PBS and fixed with 0.5% glutaraldehyde in PBS (pH 7.2) for 5 min at room temperature. Cells were then washed with PBS containing MgCl_2_ (pH 7.2, 1 mM MgCl2) and stained in X-gal solution (1 mg/ml X-gal, 0.12 mM K_3_Fe[CN]_6_(Potassium Ferricyanide), 0.12 mM K_4_Fe[CN]_6_(Potassium Ferrocyanide), 1 mM MgCl2 in PBS, pH 6.0) overnight at 37°C. The cells were washed twice with PBS, and images were captured with a microscope (IX70, Olympus, Japan).

### Western blot analysis

Western blot analyses of DNMT1, DNMT3a, DNMT3b, BMI1, EZH2, p16^Ink4A^, p21^WAF1/Cip1^, CDK2, CDK4 and β-actin were performed as described previously [39]. hUCB-MSCs cultured with or without 5-AzaC(Sigma, USA) inhibitors for 1, 3, 5 or 7 d were lysed with 50 mM Tris-HCl buffer containing 0.1% Triton X-100 freshly supplemented with a protease/phosphatase inhibitor cocktail. Proteins were then separated using 7.5–15% SDS-PAGE and transferred to nitrocellulose membranes at 350 mA for 5 h. Primary antibodies used to detect each proteins are DNMT1(polyclonal, BD, 1∶1000), DNMT3A(polyclonal, Millipore, 1∶1000), DNMT3B(polyclonal, Abcam, 1∶1000), BMI1[1.T.21](monoclonal, Abcam, 1∶1000), EZH2[BD43](monoclonal, Millipore, 1∶1000), p16^Ink4A^(polyclonal, Abcam, 1∶1500), p21^WAF1/Cip1^[CP74](monoclonal, Millipore, 1∶1000), CDK2(polyclonal, Cell-signaling, 1∶2000), CDK4[DCS156](monoclonal, Cell-signaling, 1∶2000) and β-actin[8H10D10](monoclonal, Cell-signaling, 1∶5000). All antibodies were used according to the manufacturer's instructions, and protein bands were detected using an enhanced chemiluminescence detection kit (Amersham Pharmacia Biotech, UK).

### RT-PCR

Total cellular RNA was extracted from cells with TRIzol reagent™ (Invitrogen, USA), according to the manufacturer's instructions. cDNA was synthesized by adding the purified RNA and oligo-dT primers to Accupower RT premix (Bioneer, Korea), according to the manufacturer's instructions. PCR was conducted using Accupower PCR premix (Bioneer, Korea). The primer sets sequences used for this study are supplied in Table S1. All PCR products were analyzed by gel electrophoresis on 1.5% agarose gels with ethidium bromide staining, followed by fluorescence digitization using a Bio-Rad GelDoc XR system (Bio-Rad, USA). Semi-quantitative RT-PCR was conducted by quantifying the RT-PCR bands using ImageJ image analysis software (National Institutes of Health, USA). Each gene was normalized against RPL13A as a housekeeping gene control. At least three independent analyses were carried out for each gene.

### Real-time quantitative PCR

Real-time qPCR were performed using SYBR® Green (Applied Biosystems, USA), according to the manufacturer's protocol. RPL13A was used as an internal control. All amplicons were analyzed using Prism 7000 sequence detection system 2.1 software (Applied Biosystems, USA).

### Methylation-specific PCR

For methylation-specific PCR, genomic DNA was extracted from cells with Accuprep®(Bioneer USA) according to the manufacturer's instructions. Bisulfite conversion of genomic DNA was performed using the MethyCode™(Invitrogen USA) according to the manufacturer's instructions. The sodium bisulfite-modified DNA was amplified using Accupower PCR premix (Bioneer, USA). The primers used for each promoter were designed through online web site (www.urogene.org/methprimer/) and primer sequences were supplied in Table S3.

### siRNA, anti-miRNA and mature miRNA transfection study

Transient transfection assays were performed using commercially available specific siRNAs for inhibition of DNMT1 and DNMT3b along with a non-targeting siRNA (ON Target plus SMART pool, Dharmacon, USA). Inhibition or overexpression of miRNAs was achieved by commercial antisense miRNAs or mature miRNAs of hsa-miR-200c and hsa-miR-214 with an appropriate miRNA precursor-negative control (mature miRNA: Invitrogen, USA, anti-miRNA inhibitor: Ambion, USA, and miRNA precursor-negative control #1, Ambion, USA). The siRNA, anti-miRNA and mature miRNA transfections were done according to the manufacturer's instructions. In brief, cells were seeded at a concentration of 2×10^4^/well, and siRNA-containing medium (without the addition of antibiotics) was added when the cells reached 50–60% confluence. Cells were incubated with 50 nM siRNA, 50 nM anti-miRNAs or 50 nM mature miRNAs for 48 h or 96 h. To investigate the long-term effects of inhibition, the cells were subcultured for 48–72 h after siRNA, anti-miRNA or mature miRNA transfection. Subcultured cells were stabilized for 24 h and incubated with siRNA, anti-miRNA or mature miRNA for 48–72 h at the same concentration. After inhibition, RNA extraction and subsequent RT-qPCR or SA β-gal staining was performed for genetic or characteristic analyses, respectively.

### In vitro differentiation assay

In vitro differentiation into osteogenic, adipogenic and lineages was performed as described previously [40], [41]. Briefly, hUCB-MSCs were initially cultured in growth medium containing various concentrations of 5-AzaC and then shifted to adipogenic medium (DMEM supplemented with 5% FBS, 1 µM dexamethasone, 10 µM insulin, 200 µM indomethacin and 0.5 mM isobutylmethylxanthine) or to osteogenic medium (DMEM supplemented with 5% FBS, 50 µM L-ascorbate-2-phosphate, 0.1 µM dexamethasone and 10 mM glycerophosphate). Intracellular lipid accumulation as an indicator of adipogenic differentiation was visualized by oil red O staining. After being photographed, the oil red O was eluted with 100% isopropyl alcohol and quantified with an ELISA plate reader(EL800, Bio-Tek Instruments) at OD500. Osteogenic differentiation was noted by positive staining with alizarin red S, which is specific for calcium. Neural induction was performed as described by Jori et al, with modifications [41], [42]. Briefly, hUCB-MSCs were initially cultured in pre-induction medium composed of DMEM, 5% FBS, 10 ng/ml basic fibroblast growth factor (bFGF) and HDAC inhibitors. Cells were rinsed with PBS and shifted to the neuronal induction medium consisting of 100 µM butyrated hydroxyanisole (BHA), 50 µM forskolin, 2% dimethyl sulphoxide, 25 mM KCl, 2 mM valproic acid, 2%B27 supplement(Gibco BRL), 10 ng/ml basic fibroblast growth factor(bFGF) and 10 ng/ml platelet-derived growth factor(PDGF) in a base of DMEM. Cells were maintained in induction medium for up to 24 h.

### Immunocytochemistry

Immunocytochemical analyses of TUJ1 were performed. Cells were cultured in neural pre-induction media with or without 5-azaC. Neural induction was performed after 1day pre-induction and fixed in 4% paraformaldehyde and permeabilized with 0.2% Triton X-100 (Sigma Aldrich, USA). The cells were then incubated with 10% normal goat serum (Zymed Laboratories Inc., USA) and stained with antibodies against TUJ1 (1∶200, Abcam, UK), followed by incubation for 1 h with an Alexa 488-labeled secondary antibody (1∶1000; Molecular Probes, USA). The nuclei were stained with Hoechst 33258 (1 µg/ml; 10 min), and images were captured with a confocal microscope (Eclipse TE200, Nikon, Japan).

### Chromatin immunoprecipitation (ChIP) assays

The hUCB-MSCs were seeded in 10-cm plates at a density of 0.8–1×10^5^ per plate and cultured with or without 5-azaC for 1 or 3 d. ChIP assays were performed according to the manufacturer's protocol (ChIP assay kit, Upstate Biotechnology, USA). Chromatin was immunoprecipitated using antibodies, according to the manufacturer's instructions. Real-time qPCR was performed at a final template dilution of 1∶50. The primer sequences used in the ChIP assays in this study are supplied in Table S2.

### Measurement of proliferation potential and cell cycle distribution

The effects of cellular senescence or 5-AzaC on MSC proliferation were measured using the 3-(4,5-dimethylthiazol-2-yl)-2,5-diphenyltetrazolium bromide (MTT, Sigma-Aldrich, USA) assay as described previously [39]. In brief, cells were plated on 24-well plates at a density of 2×10^4^/ml and cultured with or without 5-AzaC for 1,2 or 3days. At the end of the incubation, 50 µl of MTT stock solution (5 mg/ml) was added, and the plates were incubated for another 4 h at 37°C. Formazan crystals were solubilized with DMSO, and the absorbance was measured with an EL800 microplate reader (BIO-TEK Instruments, USA).

Flow cytometry cell cycle analysis using propidium iodide staining was also performed as previously described [21]. Briefly, MSCs in exponential growth phase were treated with HDAC inhibitors for 3 days and then harvested by trypsinization. Cells were washed with ice-cold PBS and then fixed with 70% ethanol at −20°C and stained with 50 µg/ml of propidium iodide in the presence of 100 µg/ml RNase A for 30 min. Cell cycle distribution was analysed using the FACSCalibur system (Becton Dickinson, Franklin Lakes, NJ, USA).

### Statistical analysis

All experiments were conducted at least in triplicate (n = 3), and results are expressed as the mean ± SD. Statistical analysis was conducted via analysis of variance (ANOVA), followed by Student's *t*-test. *p*<0.05 was considered to be significant.

## Results

### Replicative senescence of human MSCs

**Figure 1:**
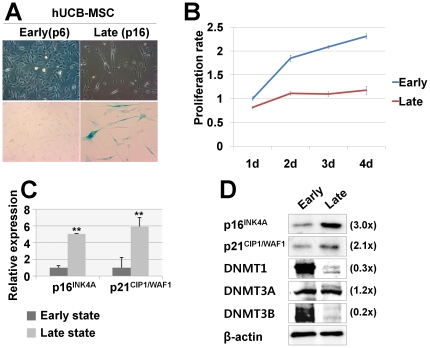
Replicative senescence of hUCB-MSCs. (a) MSCs undergo replicative senescence upon repeated (more than 15 passages) subculturing *in vitro*, as shown by SA β-gal staining. (b) Proliferation rates of MSCs in early and late passages were measured by MTT assay. (c–d) The expression of DNMT1, DNMT3A and DNMT3B was down-regulated, whereas p16^INK4A^ was up-regulated during repeated subculture-induced senescence of MSCs, as shown by real-time qPCR (c) and immunoblot analysis (d). * and ** represent statistical significance at the levels of *p<0.05* and *p<0.01*, respectively.

To characterize cellular senescence in human MSCs, we induced replicative senescence of hUCB-MSCs and human adipose tissue -derived multipotent stem cells (hAD-MSCs) by repeated sub-culture. A definite phenotype of cellular senescence was confirmed in hUCB-MSCs at passages higher than 15 (p15), as shown by SA β-gal staining (Fig. 1a and Fig. S1a). A remarkable difference in the cellular proliferation rate between p6 and p16 was confirmed by an MTT assay (Fig. 1b) and cell cycle progression between p6 and p13 was confirmed by FACS analysis (Fig. S2). Based on these data, we hereafter refer to p6–p7 and p15–p16 in the following experiments as the early and late state, respectively. To investigate the changes in expression levels of epigenetic modifying enzymes, DNMTs were analyzed by real-time PCR and western blot analysis. Both mRNA and protein levels of CDK inhibitors, p16^INK4A^ and p21^CIP1/WAF1^ were increased. However, DNMT1 and DNMT3b were decreased in the senescent hUCB-MSCs (Fig. 1c and 1d), and there was no significant change in DNMT3a expression levels during senescence. hAD-MSCs also showed similar pattern of gene expressions with hUCB-MSCs (Fig. S3).

### DNMT inhibition induces cellular senescence, cell cycle arrest and decreased multipotency

**Figure 2:**
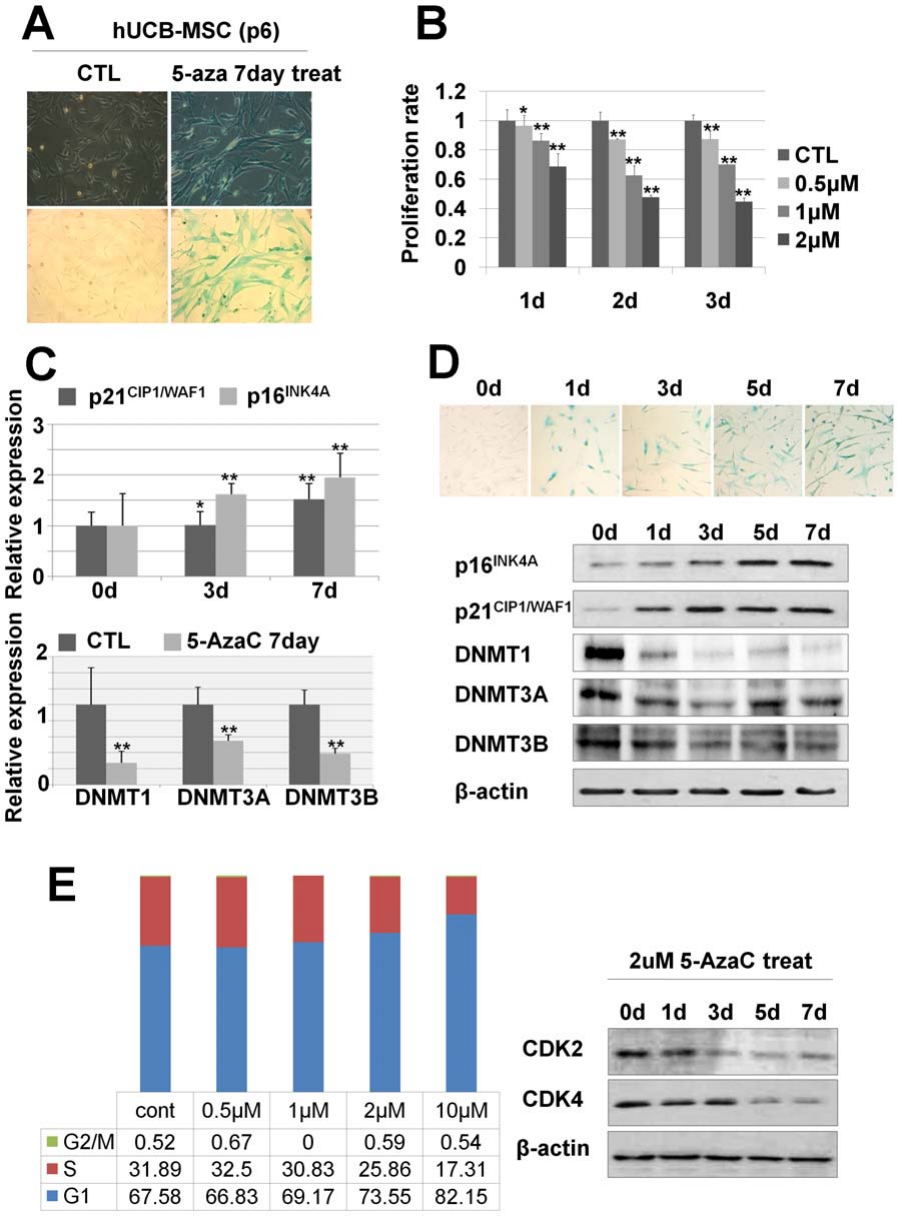
DNMT inhibition induced cellular senescence. (a) hUCB-MSCs were treated with the DNMT inhibitor 5-AzaC for 7 days. DNMT inhibition by 5-AzaC induced cellular senescence, as shown by SA β-gal staining. (b) After 5-AzaC treatment for 1, 2 and 3 days, an MTT assay was performed. (c–d) 5-AzaC increased p16^INK4A^ and p21^WAF1/Cip1^ and decreased DNMT1, DNMT3A and DNMT3B, as shown by real-time qPCR analysis (c) and western blot analysis (d). 5-AzaC treatment for 1, 3, 5 and 7 days induced cellular senescence of hUCB-MSCs, as shown by SA β-gal staining (d). (e) After a 2 day treatment with 5-AzaC, FACS analysis was performed, as described in the Materials and Methods section. 5-AzaC treatment induced G1 phase cell cycle arrest in a dose-dependent manner. CDK2 and CDK4 expression levels were confirmed by western blot analysis.

During spontaneous cellular senescence of hUCB- and hAD-MSCs, DNMT1 and DNMT3b expression levels were decreased, and p16^INK4A^ and p21^CIP1/WAF1^ levels were increased. The over-expression of DNMTs has been reported in several cancer cell lines, and DNMT inhibitors such as 5-AzaC have been well studied. Inhibition of DNMTs decrease cellular growth and induce apoptosis of cancer cells [43], [44]. However, there are no studies explaining the relationship between DNMTs and spontaneous senescence of normal adult stem cells. In order to elucidate whether the inhibition of DNMTs could induce cellular senescence of hUCB- and hAD-MSCs, we treated hUCB- and hAD-MSCs with the DNMT inhibitor 5-azacytidine (5-AzaC) and investigated phenotypic changes in the cells. DNMT inhibition by 5-AzaC treatment induced cellular senescence, as shown by SA β-gal staining (Fig. 2a, 2d, Fig. S1b, Fig. S4a and S4c), and decreased the cellular proliferation rate in a dose-dependent manner, as shown by an MTT assay (Fig. 2b and Fig. S4b). Cellular senescence is closely related to a loss of stemness. To determine the role of DNMT on the stemness of hUCB-MSCs, we investigated multipotency after treatment with 5-AzaC. We differentiated MSCs into osteogenic, adipogenic and neural lineages after 5-AzaC treatment and found that the differentiation of MSCs to all three lineages were decreased after 5-AzaC treatment, indicating that DNMT inhibition decreased the differentiation capacity of hUCB-MSCs (Fig. S5). To investigate time-dependent phenotypic and gene expression changes, we treated hUCB- and hAD-MSCs with 5-AzaC for 1, 3, 5 and 7 days and performed SA β-gal staining, RT-qPCR and western blot analyses (Fig. 2c–2d, Fig. S1c and S4d). DNMT isoforms began to decrease at 1 day after treatment with 5-AzaC. SA β-galactosidase activity and expression levels of p16^INK4A^ and p21^CIP1/WAF1^ were increased at day 3 of 5-AzaC treatment, and prominent changes were observed after 5 days of treatment with 5-AzaC. Because p16^INK4A^ and p21^CIP1/WAF1^ are CDK inhibitors that block G1 phase progression [45], we analyzed cell cycle progression by FACS analysis after treatment of hUCB-MSCs with 5-AzaC for 2 days in various concentrations to elucidate the effects of CDK inhibitors on the cell cycle during cellular senescence induced by DNMT inhibition. The results showed that DNMT inhibition induced G1 phase arrest in hUCB-MSCs in a dose-dependent manner. CDK2 and CDK4, which are direct targets of p16^INK4A^ and p21^CIP1/WAF1^, were decreased, as shown by western blot analysis. Taken together, increased p16^INK4A^ and p21^CIP1/WAF1^ as a result of inhibition of DNMTs down-regulated CDK2 and CDK4 expression and induced G1 phase cell cycle arrest in hUCB-MSCs (Fig. 2e).

### Inhibition of DNMT1 and DNMT3b induces cellular senescence

**Figure 3:**
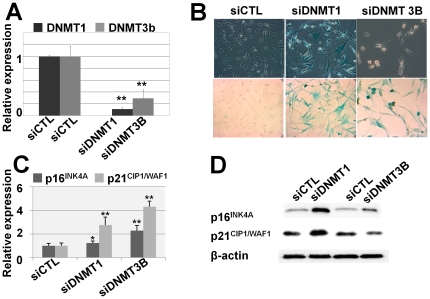
Specific inhibition of DNMT1 and DNMT3b induced cellular senescence. (a) Specific inhibition of DNMT1 and DNMT3B using siRNA was performed, as described in the Materials and Methods section. The expression levels of DNMT1 and DNMT3B were decreased, as shown by real-time PCR analysis. (b) Specific down-regulation of DNMT1 and DNMT3B caused cellular senescence in MSCs, as shown by SA β-gal staining. (c–d) The expression levels of p16^INK4A^ and p21^CIP1/WAF1^ were confirmed by real-time qPCR (c) and western blot analysis (d).

In spontaneous and 5-AzaC-induced cellular senescence, DNMT1 and DNMT3b were consistently decreased. To elucidate the effects of each DNMT isoform, inhibition of DNMT1 and DNMT3b was performed using specific siRNAs. Phenotypic and gene expression changes were investigated to confirm the reproducibility of DNMT inhibition (Fig. 3a–d). Specific inhibition of DNMT1 and DNMT3b induced cellular senescence, as shown by SA β-gal staining (Fig. 3b and Fig. S1d) and increased expression levels of p16^INK4A^. DNMT1 and DNMT3b inhibition also induced p21^CIP1/WAF1^ mRNA expression. Consistent with the results presented in Figure 2, these data showed that the inhibition of DNMT activity increases expression levels of CDK inhibitors and causes cellular senescence of hUCB-MSCs.

### DNMT inhibition modifies histone marks, transcriptional enzymes and the CpG island methylation status in the CDKi promoter regions

**Figure 4:**
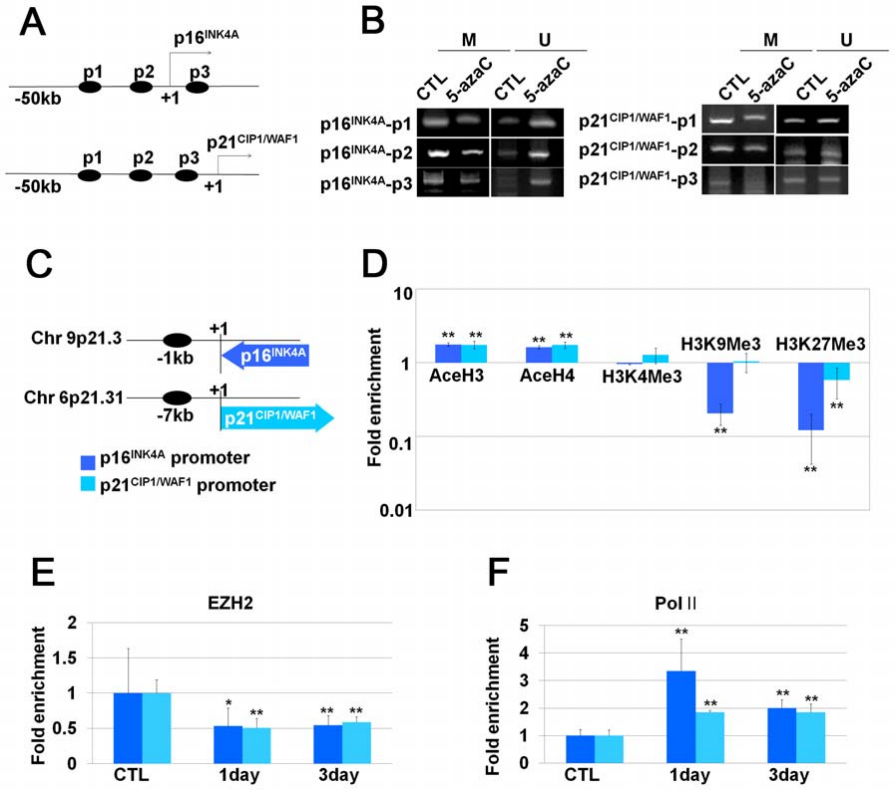
DNMT inhibition modified histone marks, transcriptional enzymes and the CpG island methylation status in the CDKi promoter regions. (a–b) After treatment with 5-AzaC for 5 days, methyl-specific PCR was performed. (a) Schematic diagrams indicate locations of each primer on CDKi promoter regions. (b) Methyl-specific PCR was performed as described in the Materials and Methods section. M: methyl primer, U: unmethyl primer. (c–f) After treatment with 5-AzaC for 3 days, ChIP analysis was performed using antibodies targeting the indicated protein (AcetylH3, AcetylH4, H3K4Me3, H3K9Me3, H3K27Me3, PolII and EZH2). (c) Schematic diagrams indicate the locations of each primer on genomic DNA. (d–f) Fold enrichment of indicated proteins on the promoters of p16^INK4A^ and p21^WAF1/Cip1^ were investigated by real-time PCR.

**Figure 5:**
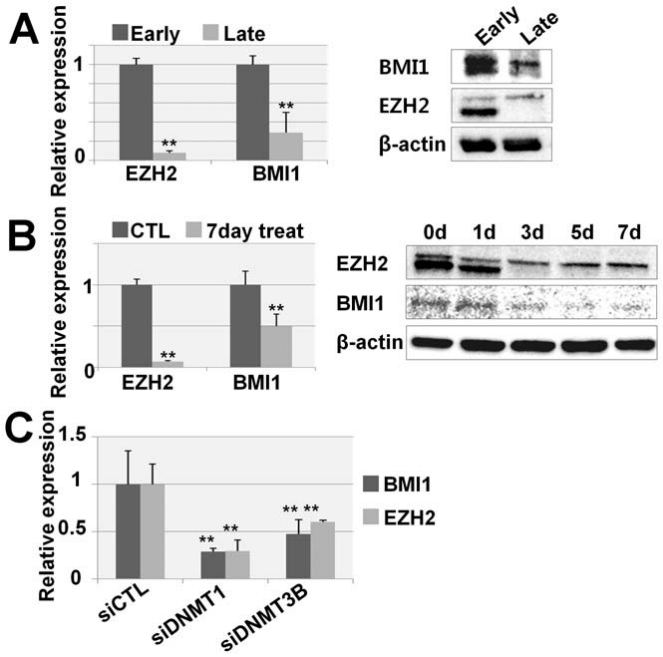
DNMT inhibition decreased PcG expression. (a) EZH2 and BMI1 expression levels were investigated by real-time qPCR (left) and western blot (right) in early and late passages of hUCB-MSCs. (b) EZH2 and BMI1 expression levels were investigated by real-time qPCR (left) and western blot (right) after the indicated duration treatment of 5-AzaC. (c) Expression levels of BMI1 and EZH2 were investigated by real-time qPCR after DNMT1 and DNMT3B inhibition.

To investigate the effect of DNMT inhibition on the epigenetic status of CDKi (p16^INK4A^ and p21^CIP1/WAF1^) promoter regions, we confirmed the methylation status of CpG islands and the changes of histone marks after 5-AzaC treatment. The CpG islands in the promoter regions of p16^INK4A^ and p21^CIP1/WAF1^ were investigated with an online web site (http://cpgislands.usc.edu/) according to the following lower limits: %GC = 50, Observed CpG/Expected CpG = 0.6, Length = 200 and Distance = 100. A total of 5 and 10 CpG islands were found within 50 kbp upstream of the promoter regions of p16^INK4A^ and p21^CIP1/WAF1^, respectively. Methylation of the CpG islands on these promoter regions was investigated after 5-AzaC treatment, and we found decreased methylation of the CpG islands. However, there were profound differences in the CpG methylation status between the p16^INK4A^ and p21^CIP1/WAF1^ promoter regions of untreated control hUCB-MSCs. The CpG islands in the p16^INK4A^ promoter region were highly methylated, as demonstrated by hardly detectable levels of unmethylated product bands (Figure 4B). However, the ratio of methylated CpG islands in the p21^CIP1/WAF1^ promoter region was approximately 50% according to the product band intensity. This result suggested that demethylation of CpG islands would have more of an effect on the regulation of p16^INK4A^ expression than p21^CIP1/WAF1^ expression (Fig. 4a and 4b) because the p16^INK4A^ promoter is more highly methylated than the p21^CIP1/WAF1^ promoter at basal levels in hUCB-MSCs. Considering that the DNA methylation status is highly related to histone modification, we also investigated the histone modification status of the p16^INK4A^ and p21^CIP1/WAF1^ promoter regions after inhibition of DNMTs by 5-AzaC treatment. We found that the active histone forms, acetyl H3 and acetyl H4, were increased. However, inactive forms of histones, such as H3K9Me3 and H3K27Me3, were decreased in the p16^INK4A^ and in p21^CIP1/WAF1^ promoter regions following inhibition of DNMTs. H3K4Me3, an active histone form, did not change following 5-AzaC treatment (Fig. 5c–d). Binding of EZH2, a polycomb protein with methyltransferase activity that methylates histone H3K9 and H3K27, were decreased. However, binding of RNA polymerase II was significantly increased on the p16^INK4A^ and p21^CIP1/WAF1^ promoter regions after 5-AzaC treatment (Fig. 5e–f). These data indicate that DNMT regulates p16^INK4A^ and p21^CIP1/WAF1^ expression levels by both direct modification of DNA methylation and indirect histone modifications on the p16^INK4A^ and p21^CIP1/WAF1^ promoter regions.

### DNMT inhibition decreased PcG expression

Considering the decreased EZH2 binding on the p16^INK4A^ and p21^CIP1/WAF1^ promoter regions, we assessed whether DNMT inhibition affects the expression levels of EZH2 and BMI1, a PRC1 protein that is recruited to the PRC2 binding site and maintains transcriptional repression. As we have previously reported, EZH2 and BMI1 expression levels were significantly decreased in replicative senescence (Fig. 5a). After inhibition of DNMT by 5-AzaC treatment, we observed decreased EZH2 and BMI1 expression levels (Fig. 5b). Specific inhibition of DNMT1 and DNMT3b with siRNA also consistently decreased expression levels of EZH2 and BMI1 (Fig. 5c).

### PcG-targeting microRNAs were upregulated after DNMT inhibition

**Figure 6:**
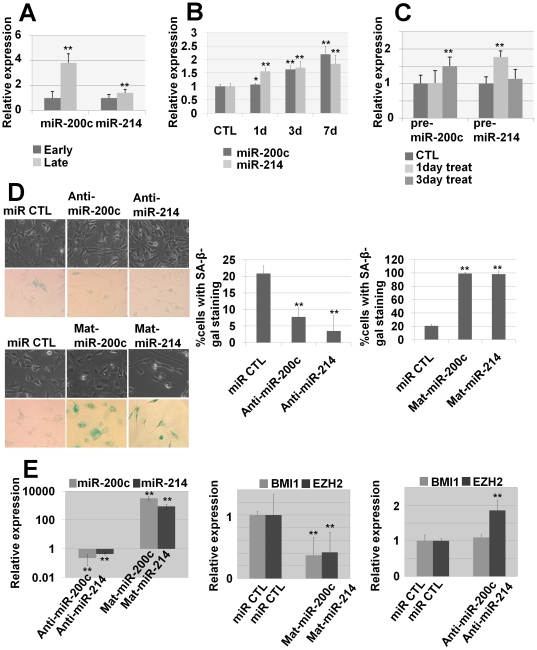
PcG-targeting microRNAs were upregulated after DNMT inhibition. (a–c) To confirm the expression levels of PcG-targeting microRNAs in early and late passage MSCs and 5-AzaC-treated MSCs, real-time qPCR analysis was performed. Relative expression levels of mature microRNA 200c and 214 in early and late passage (a) and 1, 3 and 7day, 5-AzaC-treated hUCB-MSCs (b) were visualized. Relative expression levels of precursor microRNA 200c and 214 in 1–3 day, 5-AzaC-treated hUCB-MSCs were visualized (c). (d–e) miR200c and miR-214 inhibition and overexpression studies were performed. (d) Overexpression of both miRNAs induced cellular senescence of hUCB-MSCs, as shown by SA β-gal staining. (e) After transfection of anti- and mature-miRNA oligonucleotides, the expression levels of each miRNA and EZH2 and BMI1 were evaluated by real-time qPCR.

DNMT is well known to suppress gene expression by DNA methylation. Thus, one may speculate that the effect of DNMT inhibition on p16^INK4A^ and p21^CIP1/WAF1^ expression is due to transcriptional reactivation. In this context, decreases of BMI1 and EZH2 by inhibition of DNMTs should have negative mediators, which may increase during DNMT inhibition. Considering that BMI1 and EZH2 expression is regulated at the mRNA and protein level, the mediators, if any, would regulate mRNA and/or protein expression of BMI1 and EZH2. Given that miRNAs have common inhibitory functions on gene expression by targeting mRNAs, they could be reasonable candidates as inhibitory mediators. To confirm whether miRNAs are involved in the regulation of PcG by DNMT inhibition, we observed expression levels of miRNAs in both spontaneous and 5-AzaC-induced cellular senescence. It is well known that miR-214 targets EZH2 and that miR-200c targets BMI1 [33], [46]. By real-time qPCR analysis, we confirmed that miR-200c and miR-214 were up-regulated in senescent hUCB-MSCs (Fig. 6a). Because the significant decrease of EZH2 and BMI1 occurs after 3 days of treatment with 5-AzaC, we investigated miRNA expression levels at 1, 3 and 7 days after treatment with 5-AzaC and found that both mature and precursor miRNAs were increased at the time points indicated (Fig. 6b and 6c). To confirm whether the targets of miR-200c and miR-214 are BMI1 and EZH2, respectively, in hUCB-MSCs, we performed miRNA inhibition and overexpression experiments using transient transfection of anti- and mature-miRNA oligonucleotides. After overexpression of miR-200c and miR-214, MSCs underwent cellular senescence, as shown by SA β-gal staining, and BMI1 and EZH2, the respective targets of miR-200c and miR-214 were decreased, as shown by real-time qPCR (Fig. 6d and 6e). In addition, inhibition of miR-214 using antisense oligonucleotide transfection increased EZH2 expression (Fig. 6e). However, after inhibition of miR-200c, BMI1 expression was not changed at the mRNA level. Although inhibition of miR-200c did not yield consistent results, overexpression of both miRNAs decreased their respective target (BMI1 and EZH2) at the mRNA level, suggesting that overexpressed miRNA during cellular senescence regulates the expression levels of BMI1 and EZH2.

### DNMT inhibition modifies the CpG island methylation status, histone marks and transcriptional enzymes in the vicinity of genomic DNA regions of miRNAs

**Figure 7:**
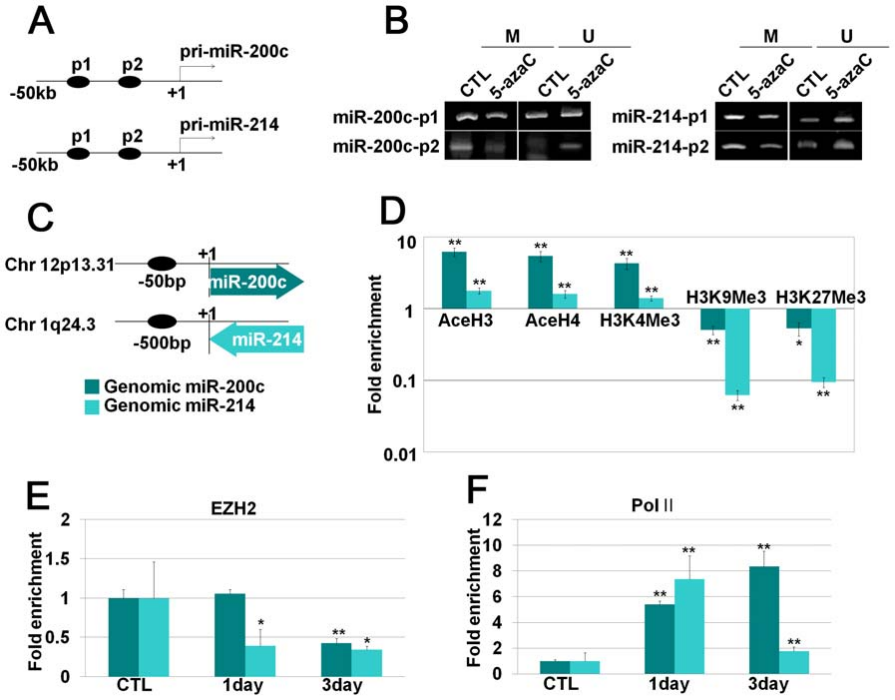
DNMT inhibition modified histone marks, transcriptional enzymes as well as the CpG island methylation status in the vicinity of miR-200c and 214 genomic regions. (a–b) After treatment with 5-AzaC for 5 days, methyl-specific PCR was performed. (a) Schematic diagrams indicate locations of each primer in the vicinity of miR-200c and -214 genomic regions. (b) Methyl-specific PCR was performed as described in the Materials and Methods section. M: methyl primer, U: unmethyl primer. (c–f) After treatment with 5-AzaC for 3 days, ChIP analysis was performed using antibodies targeting to the indicated proteins (AcetylH3, AcetylH4, H3K4Me3, H3K9Me3, H3K27Me3, PolII and EZH2). (c) Schematic diagrams indicate the locations of each primer on genomic DNA. (d–f) Fold enrichment of indicated proteins in the vicinity of miR-200c and -214 genomic regions were investigated by real-time qPCR.

According to the results shown in Figure 4, DNMT inhibition induced the expression of miR-200c and miR-214, which target PcGs. Recalling that the major function of DNMT involves epigenetic regulation, we investigated the epigenetic status of the genomic regions of miRNAs by measuring the CpG island methylation status, histone marks and the related binding proteins at the genomic regions of miRNAs. CpG islands in the vicinity of miRNAs were investigated using the previously mentioned online website. After DNMT inhibition by 5-AzaC for 3 days, methylation of CpG islands in the vicinity of miRNA genomic regions was investigated by methyl-specific PCR. We observed that methylation of CpG islands was decreased after DNMT inhibition (Fig. 7a and 7b). After DNMT inhibition by 5-AzaC for 1 and 3 days, we performed ChIP analysis followed by real-time qPCR analysis using primers against the genomic regions of miRNAs. Binding of active histone marks, acetyl histone H3 and H4 and H3K4Me3 were significantly increased in both miRNA genomic regions. However, the fold enrichment of inactive histone marks, histone H3K9Me3 and H3K27Me3 were significantly decreased in both miRNA genomic regions (Fig. 7c and 7d). Although EZH2 is a target of miR-214, EZH2 itself could be involved with the regulation of histone H3K27 methylation at miRNA genomic regions in a negative feedback manner. To confirm this, we also investigated the binding level of EZH2 to the genomic regions of miRNAs and found that EZH2 binding to miRNA genomic regions was decreased after 5-AzaC treatment (Fig. 7e). To obtain direct evidence of the transcriptional regulation of miRNA expression, RNA polymerase II (PolII) enrichment on the miRNA genomic region was investigated, and we confirmed an increase of PolII binding (Fig. 7f).

## Discussion

In this study, we determined that DNMTs regulate the cellular senescence of hUCB-MSCs by controlling the expression of p16^INK4A^ and p21^CIP1/WAF1^ through epigenetic modification. In addition to this, we also unveiled the mechanism how DNMT regulates BMI1 and EZH2 by controlling the expression of miRNAs during cellular senescence.

We found that the DNMT isoforms DNMT1 and DNMT3B were decreased during the cellular senescence of hUCB-MSCs. Stem cells and cancer cells are able to expand while maintaining undifferentiated properties. Although it is well known that the over-expression of DNMT suppresses p16^INK4A^ in various cancer cells, the regulatory roles of DNMT on stem cell aging and self-renewal have not been well studied. There are two studies showing the involvement of DNMTs in normal human fibroblast aging [47], [48]. In this study, we first clarified the involvement of DNMT on the cellular senescence of hUCB-MSCs. The inconsistent changes in expression levels among the DNMT isoforms, as shown in fig. 2b, have already been reported in several studies. Chen, C. et al. showed that DNMT1 and DNMT3B mRNAs were overexpressed, although DNMT3A expression was not changed in primary and recurrent epithelial ovarian carcinoma [41]. Datta, J et al. demonstrated that Dnmt3b and Dnmt1 make a co-repressor complex that exhibits *de novo* DNA methyltransferase activity. Dnmt3a is related to Hdac1 and HMTase activity [49], [50]. Vinken, M. et al. reported that DNMT3A was decreased during Fas-mediated hepatocyte apoptosis, whereas DNMT1 and DNMT3B showed no changes [51]. According to our results, decreases in DNMT1 and DNMT3B were associated with spontaneous senescence of hUCB-MSCs, but DNMT3A was not. Specific inhibition of both DNMT1 and DNMT3B increased p16^INK4A^ expression and SA β-gal activity. However, in DNMT3B-inhibited cells, some apoptotic cell death was observed. Considering that DNMT inhibition by 5-AzaC did not cause apoptosis, the extent of DNMT3B inhibition could have shifted cellular senescence to apoptosis. Another possibility is that DNMT3B inhibition alone induces apoptosis, but the overall down-regulation of DNMT isoforms could induce cellular senescence through another pathway.

Inhibition of DNMTs increased the expression levels of CDK inhibitors p16^INK4A^ and p21^CIP1/WAF1^, followed by G1 phase cell cycle arrest, a decreased cell proliferation rate and an induction of cellular senescence. Osteogenic, adipogenic and neural differentiation abilities of MSCs were also decreased after DNMT inhibition. In addition, MSCs are able to differentiate into myogenic lineage [52], [53], [54], [55], [56], [57], [58], [59]. It was reported that epigenetic modifying drugs induces nonmesenchymal differentiation. Valproic acid, a HDAC inhibitor was used for neural induction of MSCs [60], [61], and 5-AzaC is a well known inducer of myogenic differentiation of MSCs [54], [55], [56], [57], [58], [59]. There are a number of studies that report DNMT inhibition causes bone marrow derived multipotent progenitor cells and embryonic stem cells to differentiate into endothelial cells [62], [63]. Taken together, 5-AzaC has decreased the differentiation potential of hUCB-MSCs into adipogenic and osteogenic lineages as well as neuronal cells in the present study. Because we did not examine whether 5-AzaC affects myogenic and endothelial differentiation of hUCB-MSCs, there are still possibilities that the role of 5-AzaC in MSC differentiation is cell type specific. This would be worthy of further research to extend our understandings of regulation mechanisms of MSC differentiation.

In the present study, we first elucidated how DNMT regulates p16^INK4A^ and p21^CIP1/WAF1^ and induces cellular senescence of hUCB-MSCs. According to our results, DNMT inhibition induced histone modulation and decreased DNA demethylation at the p16^INK4A^ and p21^CIP1/WAF1^ promoter regions. As methylated DNA is bound by methyl-CpG binding protein (MeCP) complexes that include HDACs, DNA demethylation followed by histone acetylation on the promoter regions after DNMT inhibitor treatment is reasonable. According to one report, DNMT3A is associated with HDAC1 and HMTase, suggesting that DNMT3A could be one of the mediators bridging DNA methylation and histone acetylation/methylation. Decreases in both EZH2 expression levels and EZH2 enrichment at the p16^INK4A^ and p21^CIP1/WAF1^ promoter regions supports our ChIP results, which showed the demethylation of H3K9Me3 and H3K27Me3 during cellular senescence. In our supplementary data (Fig. S6), KDM2B (histone H3K4 demethylase) was decreased and JMJD3 (histone H3K27 demethylase) was increased in the replicative or DNMT inhibitor-induced senescent state when compared to early passage or control cells. Considering that the expression levels of genes reflect their global activity in the cells, these changes in histone demethylase expression levels also support the changes in histone H3K4 or H3K27 methylation on promoter regions, which were investigated in this study. In the case of DNA methylation, according to the results of the methyl-specific PCR in the present study, the p16^INK4A^ promoter region was more methylated than the p21^CIP/1WAF1^ promoter region. As a consequence, the demethylation of of p16^INK4A^ promoter region occurred more highly than that of p21^CIP/1WAF1^ after DNMT inhibitor treatment.

In fact, the reported regulatory mechanisms of p16^INK4A^ and p21^CIP1/WAF1^ vary according to the cell line studied. In various cancer cell lines, the DNMT isoforms DNMT1 and DNMT3B are up-regulated, and as a result, the promoter region of p16^INK4A^ is hypermethylated. In this case, inhibition of DNMT up-regulates the expression level of p16^INK4A^ by controlling the DNA methylation status at the p16^INK4A^ promoter region [8], [10], [11]. Another mechanism that regulates p16^INK4A^ expression is histone modulation, as reported in several studies. In a previous study, we have reported that HDAC inhibition caused increased p16^INK4A^ expression levels, followed by demethylation of histone H3K27me3, which is a repressive histone mark regulated by a balance of the expression levels between PcGs and JMJD3 [4]. There are several studies that suggest that p16^INK4A^ expression is also regulated by histone acetylation. Zhou R et al. reported that p16^INK4A^ expression could be regulated by the recruitment of HDACs in human fibroblasts. Histone acetylation is a major mechanism for p21^CIP/1WAF1^ regulation in gastric cancer cell lines [64]. However, the DNA methylation status of the p21^CIP/1WAF1^ promoter region and the involvement of DNMT in the regulatory mechanism are different among cell lines. In Rat-1 cells and rhabdomyosarcomas, increased methylation at p21^CIP/1WAF1^ promoter regions has been reported. However, several studies indicated that the hypermethylation of p21^CIP/1WAF1^ was not the main mechanism by which p21^CIP/1WAF1^ expression was being regulated [65], [66]. Although Young et al. reported that DNMT inhibition caused cell cycle arrest and p21^CIP/1WAF1^ overexpression in normal human fibroblasts [66], Milutinovic et al. showed that inhibition of DNMT resulted in the rapid induction of p21^CIP/1WAF1^ without involvement of DNA demethylation in the p21^CIP/1WAF1^ promoter in the A549 human non-small cell lung cancer cell line [67]. Shin et al. reported that the promoter of the p21^CIP/1WAF1^ gene was not methylated in gastric cancer cells. This demonstrates that the inactivation of p21^CIP/1WAF1^ in gastric cancer cells might occur independently of the DNA methylation status of the p21^CIP/1WAF1^ promoter region [64].

DNMT inhibition by 5-AzaC and specific siRNAs induced cellular senescence, followed by a decrease in BMI1 and EZH2. To date, there is no report that shows the role of DNMT in the regulation of BMI1 and EZH2. Instead, some studies have shown that BMI1, EZH2 and DNMT work together to repress gene expression by histone modulation or DNA methylation [18], [19]. We first elucidated that DNMT was associated not only with the functional activities of PcG but also with BMI1 and EZH2 expression. Regulation of miRNAs that target BMI1 and EZH2 by DNMT during cellular senescence is also a novel finding in this study. MiR-200c and miR-214 were previously reported to target BMI1 and EZH2, respectively, in studies that showed that miR-214 targets Ezh2 in skeletal muscle and embryonic stem cells and that miR-200c targets BMI1 in breast cancer stem cells [33], [46]. We confirmed that miR-200c and miR-214 were up-regulated in senescent hUCB-MSCs. Next, to confirm whether miR-200c and miR-214 regulate BMI1 and EZH2 expression levels in hUCB-MSCs, we performed transient transfection of antisense or mature miRNA oligonucleotides. The results showed that anti- and mature-miR-214 regulated EZH2 expression at the mRNA level. Transfection of mature miR-200c also decreased BMI1 expression. However, anti-miR-200c did not regulate BMI1 mRNA expression levels. Because the absolute quantity of miR-200c was relatively lower than that of miR-214 in early passages of hUCB-MSCs and expression of miR-200c would be in an inhibited state in early passage cells compared to senescent cells, additional miR-200c inhibition may have no effect on BMI1 expression. To confirm this hypothesis, additional miRNA inhibition studies should be performed in senescent cells in which miRNAs are in an up-regulated state.

Considering that DNMT is an epigenetic modulator of transcriptional activity, we questioned whether the miRNAs were regulated by transcriptional reactivation. The increase of precursor miRNAs during cellular senescence supported this possibility. To answer this question, we investigated the DNA methylation status and histone modulation of the miRNA regions after treatment of 5-AzaC. A decrease in the DNA methylation status was observed in the vicinity of the miRNA genomic region after 5-AzaC treatment. In addition, DNMT inhibition increased active histone forms, acetyl histone H3 and H4, histone H3K4Me3 and decreased H3K9 trimethylation (H3K9Me3) and H3K27Me3 in the proximity of the genomic region of miRNAs. A significant increase in RNA polymerase II bound on the indicated locations shows that the transcriptional activities might be increased at both miR-200c and miR-214 genomic regions. A decrease in EZH2 binding to the genomic region of miRNAs indicated that these miRNAs and their target PcGs affect each other reciprocally. Recently, it has been uncovered that the genomic regions of miRNAs, which act as tumor suppressors, are hypermethylated in several cancer cells as a consequence of the epigenetic regulation of miRNAs and are emerging as a significant topic in the cancer cell field [68], [69]. MiR-200c, which targets BMI1, is one of the important miRNAs that are epigenetically repressed in breast cancer cells. This study shows that miR-200c and miR-214 are related not only to cancer cells but also to the cellular senescence of MSCs, linking DNA methylation and PcG-related histone modification.

**Figure 8:**
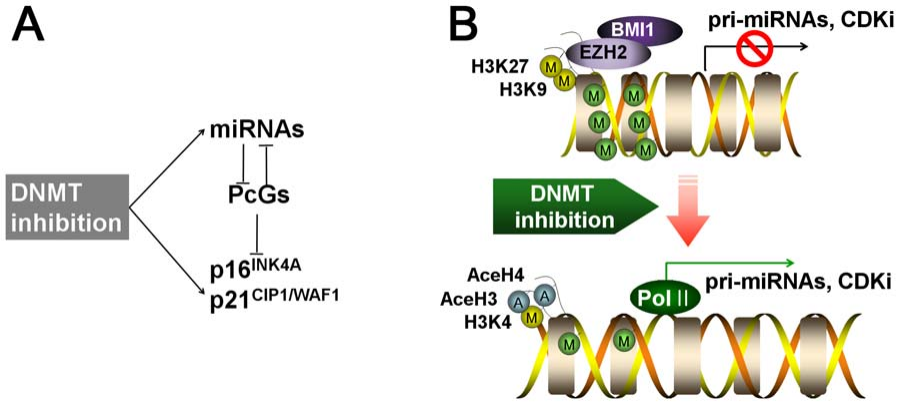
Schematic diagram describing the relationship between miRNAs, PcGs, p16 and p21 and how transcriptional regulation of miRNAs, p16^INK4A^ and p21^CIP1/WAF1^ occurs in DNMT inhibitor-mediated senescent MSCs. (a) DNMT inhibition increases p16^INK4A^ and p21^CIP1/WAF1^ expression directly through DNA demethylation, indirectly through an unknown pathway and, over time, induces cellular senescence. The regulation of miRNAs, which target PcG proteins, is one of the indirect pathways that increase p16^INK4A^ and p21^CIP1/WAF1^ expression. (b) DNMT inhibition induces CpG island demethylation, increases active histone forms and decreases inactive histone forms in the promoter region of CDK inhibitors and in the proximity of miRNAs in hUCB-MSCs. Pri-miRNA refers to primary-miRNA.

Taken together, we first report here that DNMTs have a critical role in regulating cellular senescence of hUCB-MSCs through controlling PcG-targeting miRNAs and p16^INK4A^ and p21^CIP1/WAF1^ expression epigenetically as summarized in Fig. 8. The regulation of miRNAs by DNMT enables us to explain how PcGs are downregulated after DNMT inhibition and suggest another linking mechanism between DNMT inhibition and histone modification. Extending this study, it would be meaningful to find up-regulated miRNAs, which target down-regulated proteins, and confirm their epigenetic changes after DNMT inhibition.

## Supporting Information

Figure S1 **Quantification of SA-beta-gal positive cells.** SA-beta-gal positive cells were quantified through counting cells in at least 3 microscope images for each group and presented in graph(a; Fig 1a, b;Fig 2a, c;Fig 2d, d;Fig 3b).
(TIF)

Figure S2 **Cell cycle analysis of early and late passaged hUCB-MSCs.** To compare cell cycle status of early and late passaged hUCB-MSCs, FACS analysis was performed, as described in the Materials and Methods section.
(TIF)

Figure S3 **Changes of gene expression levels in senescent hAD-MSCs.** Primary culture and long term culture of hAD-MSCs were performed as described in Methods S1. The expression of DNMT1, DNMT3A, DNMT3B, BMI1 and EZH2 was down-regulated, whereas p16^INK4A^ was up-regulated during repeated subculture-induced senescence of hAD-MSCs, as shown by RT-PCR. We quantified the results of RT-PCR analyses by using image analysis software (imageJ) and values presented as graph. * and ** represent statistical significance at the levels of *p<0.05* and *p<0.01*, respectively.
(TIF)

Figure S4 **DNMT inhibition induced cellular senescence of hAD-MSCs.** hAD-MSCs were treated with the DNMT inhibitor 5-AzaC. DNMT inhibition by 5-AzaC induced morphological change and cellular senescence, as shown by SA β-gal staining. (a, c) After 5-AzaC treatment for 3 days, an MTT assay(b) and realtime qPCR analysis(d) were performed.
(TIF)

Figure S5 **5-AzaC-induced cell cycle arrest and decreased multipotency.** Cells were pretreated with 5-AzaC for the indicated time and dose. Osteogenic, adipogenic and neural induction were performed, as described in the Materials and Methods section. (a) 5-AzaC treatment decreased the osteogenic differentiation of hUCB-MSCs, as shown by alizarin red S staining after 3 weeks of induction with osteogenic medium. RT-PCR analysis of the osteogenic marker type 1 collagen (Col-1) was performed, and semi-quantification of at least three independent assays was completed and visualized using ImageJ image analysis software. (b) After adipogenic induction, lipid droplets were visualized using oil red O staining. After being photographed, oil red O was eluted, and absorbance was measured. RT-PCR analysis of the adipogenic marker, aP2, was performed, and semi-quantification of at least three independent assays was performed and visualized using ImageJ image analysis software. (c) After 1 day of neural induction, morphological changes were observed using an inverted microscope. TUJ1 neurofilaments were visualized using immunocytochemistry, and levels of the PAX6 transcription factor, which is expressed during neurogenesis, was assessed using RT-PCR.
(TIF)

Figure S6 **Expression levels of histone demethylases in early/late passages and 5-AzaC-treated hUCB-MSCs.** RT-PCR analysis was performed to confirm the expression levels of histone demethylases, KDM2B and JMJD3.
(TIF)

Methods S1 **Isolation and culture of hAD-MSCs.**
(DOCX)

Table S1 **Primer sequences used for realtime qPCR**
(DOC)

Table S2 **Promoter primer sequences used for ChIP analysis**
(DOC)

Table S3 **Primers used for methyl specific PCR**
(DOCX)
