# Supplementary figures and images for: DNA Methyltransferase Controls Stem Cell Aging by Regulating BMI1 and EZH2 through MicroRNAs
Source: PLoS One. 2011 May 10;6(5):e19503. doi: 10.1371/journal.pone.0019503 (PMC3091856; doi:10.1371/journal.pone.0019503)

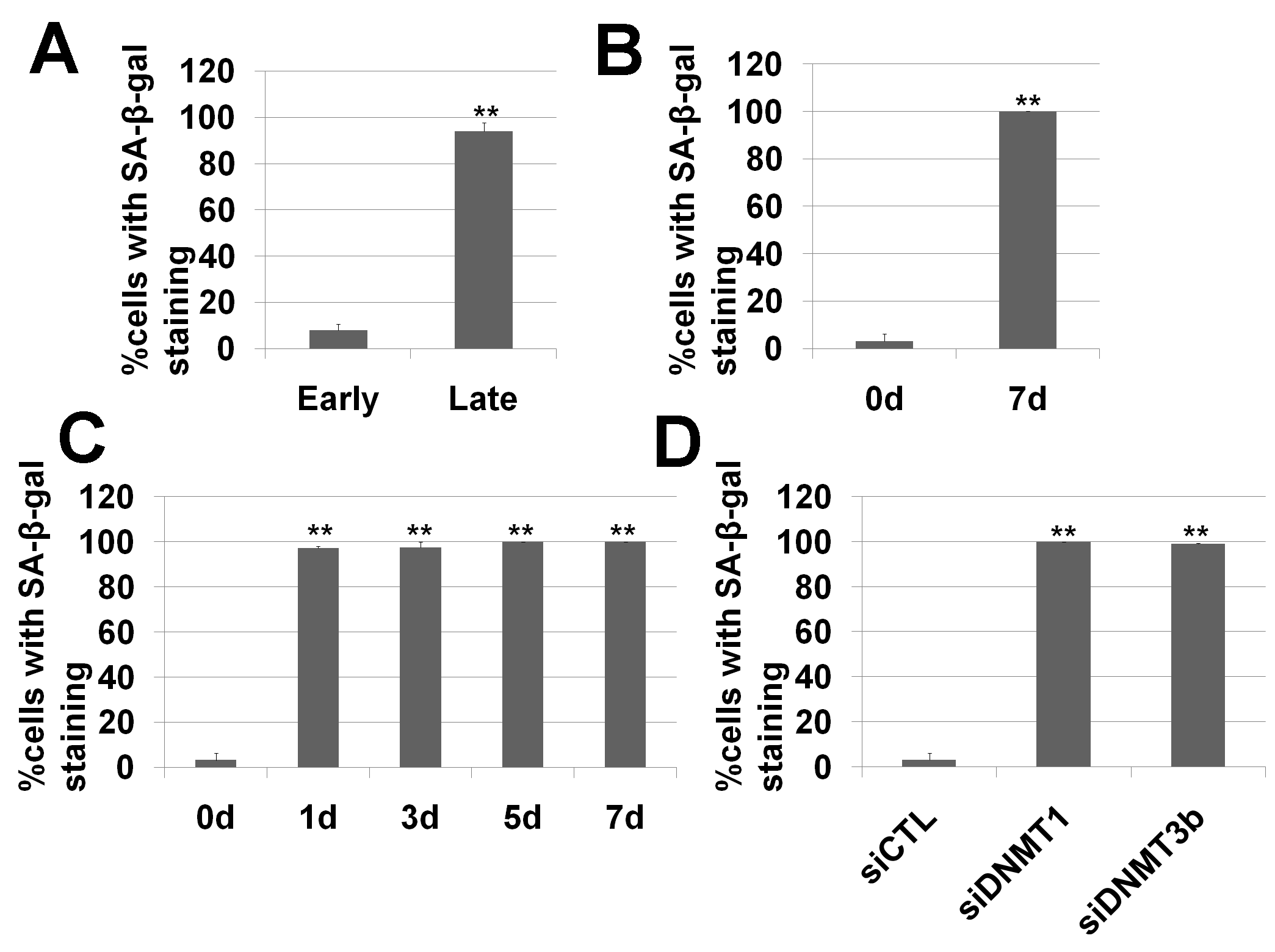

Supplement: Figure S1 — Quantification of SA-beta-gal positive cells. SA-beta-gal positive cells were quantified through counting cells in at least 3 microscope images for each group and presented in graph(a; Fig 1a, b;Fig 2a, c;Fig 2d, d;Fig 3b). (TIF) [file pone.0019503.s001.tif]

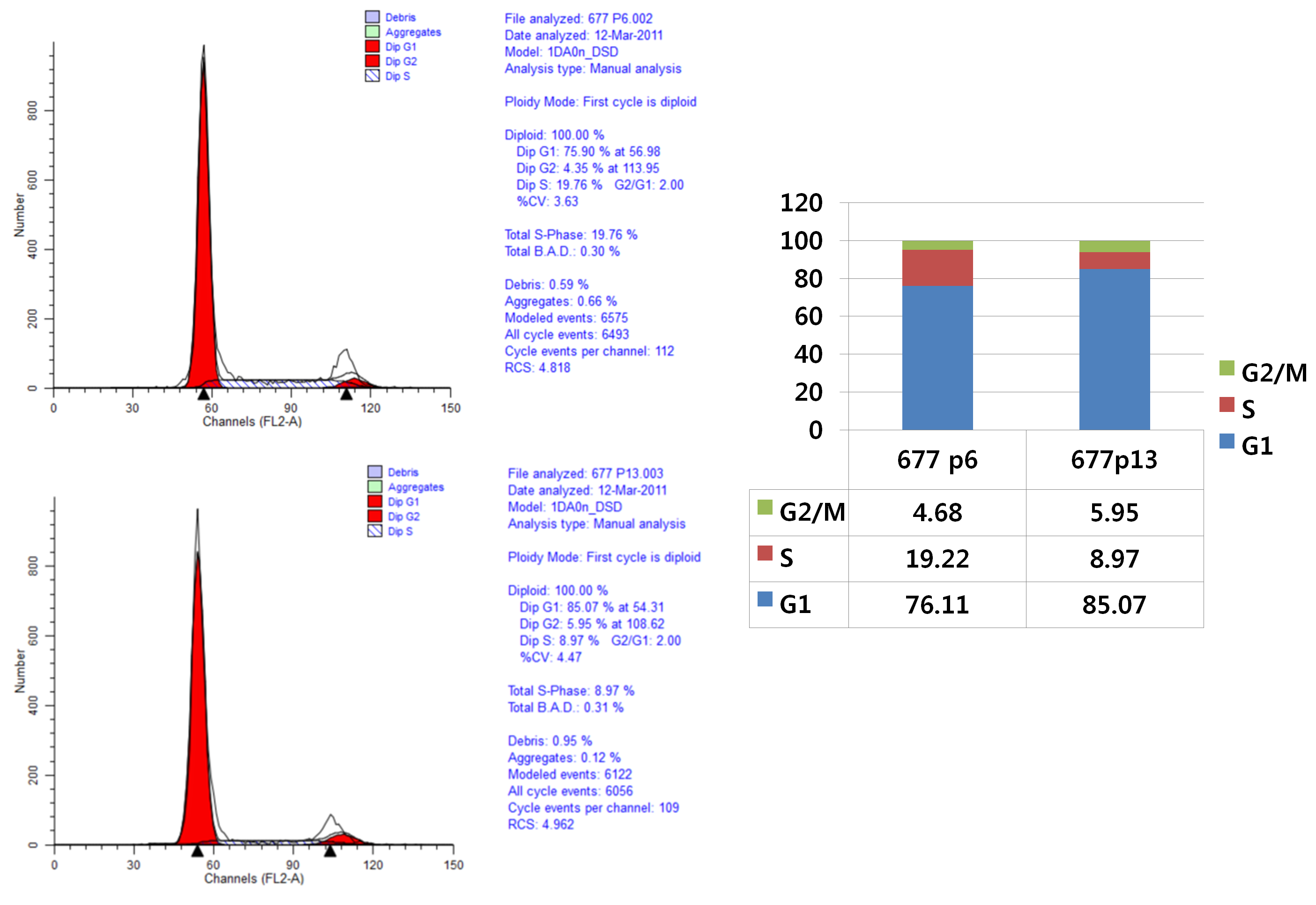

Supplement: Figure S2 — Cell cycle analysis of early and late passaged hUCB-MSCs. To compare cell cycle status of early and late passaged hUCB-MSCs, FACS analysis was performed, as described in the Materials and Methods section. (TIF) [file pone.0019503.s002.tif]

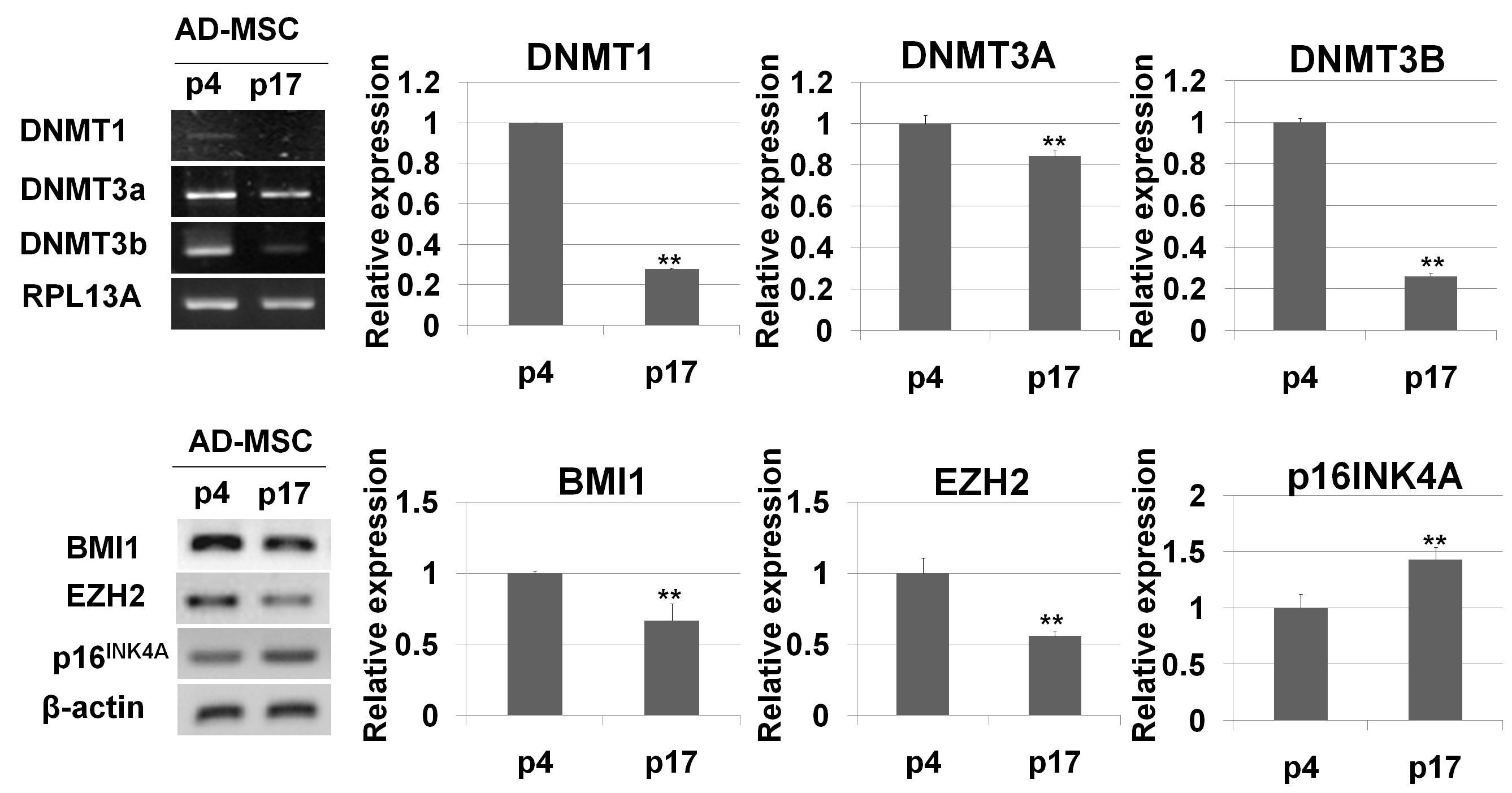

Supplement: Figure S3 — Changes of gene expression levels in senescent hAD-MSCs. Primary culture and long term culture of hAD-MSCs were performed as described in Methods S1. The expression of DNMT1, DNMT3A, DNMT3B, BMI1 and EZH2 was down-regulated, whereas p16INK4A was up-regulated during repeated subculture-induced senescence of hAD-MSCs, as shown by RT-PCR. We quantified the results of RT-PCR analyses by using image analysis software (imageJ) and values presented as graph. * and ** represent statistical significance at the levels of p<0.05 and p<0.01, respectively. (TIF) [file pone.0019503.s003.tif]

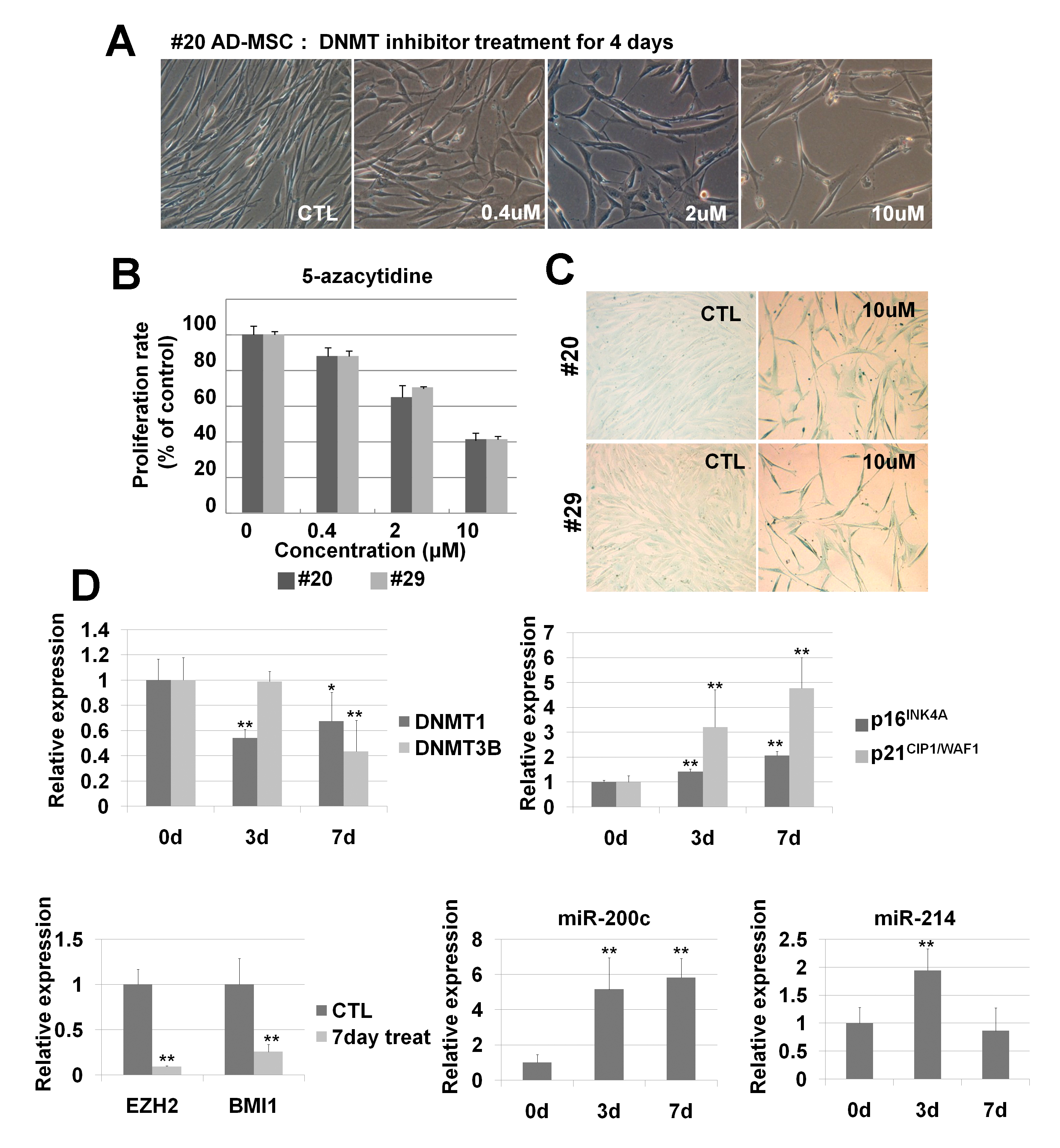

Supplement: Figure S4 — DNMT inhibition induced cellular senescence of hAD-MSCs. hAD-MSCs were treated with the DNMT inhibitor 5-AzaC. DNMT inhibition by 5-AzaC induced morphological change and cellular senescence, as shown by SA β-gal staining. (a, c) After 5-AzaC treatment for 3 days, an MTT assay(b) and realtime qPCR analysis(d) were performed. (TIF) [file pone.0019503.s004.tif]

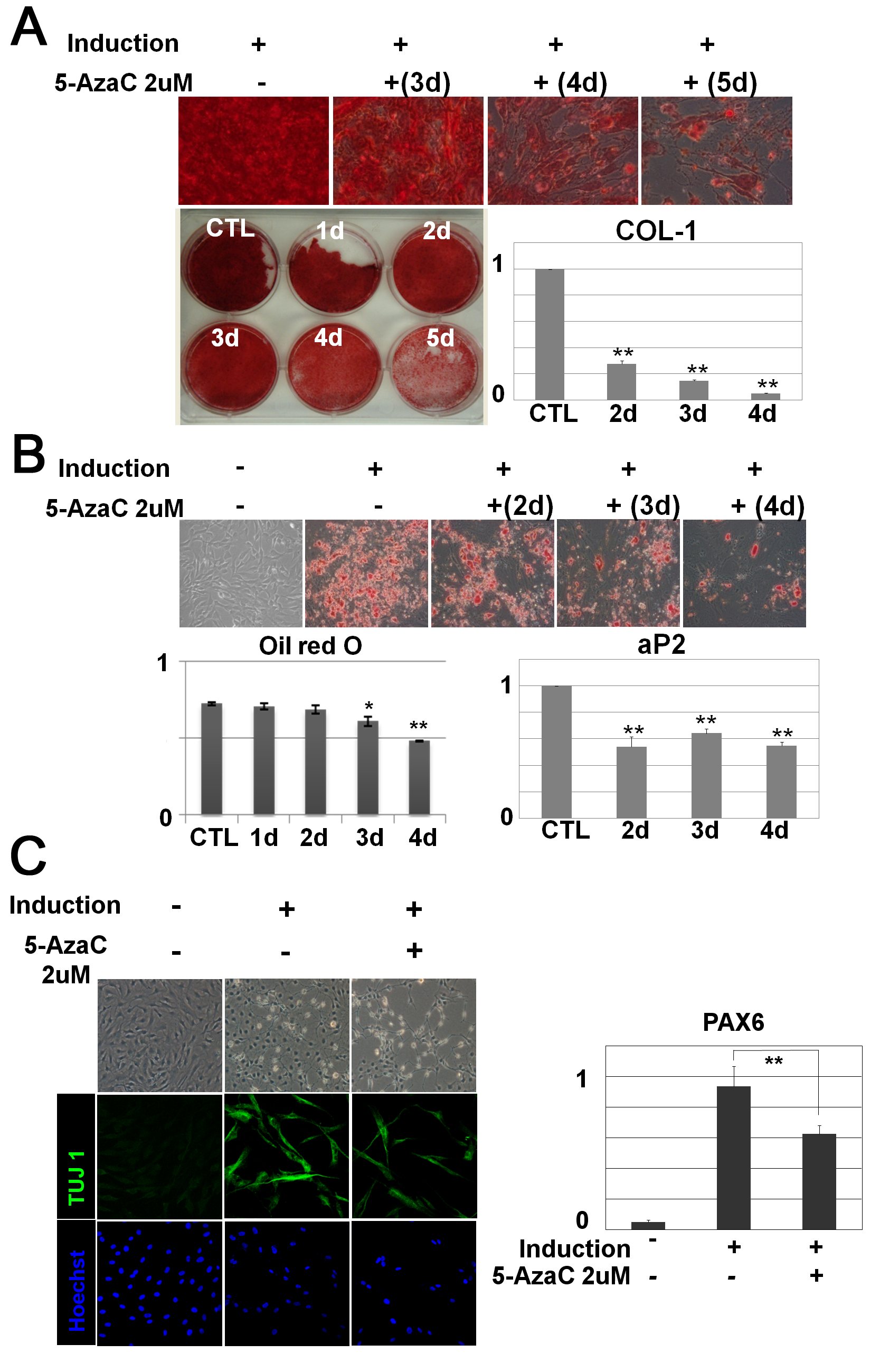

Supplement: Figure S5 — 5-AzaC-induced cell cycle arrest and decreased multipotency. Cells were pretreated with 5-AzaC for the indicated time and dose. Osteogenic, adipogenic and neural induction were performed, as described in the Materials and Methods section. (a) 5-AzaC treatment decreased the osteogenic differentiation of hUCB-MSCs, as shown by alizarin red S staining after 3 weeks of induction with osteogenic medium. RT-PCR analysis of the osteogenic marker type 1 collagen (Col-1) was performed, and semi-quantification of at least three independent assays was completed and visualized using ImageJ image analysis software. (b) After adipogenic induction, lipid droplets were visualized using oil red O staining. After being photographed, oil red O was eluted, and absorbance was measured. RT-PCR analysis of the adipogenic marker, aP2, was performed, and semi-quantification of at least three independent assays was performed and visualized using ImageJ image analysis software. (c) After 1 day of neural induction, morphological changes were observed using an inverted microscope. TUJ1 neurofilaments were visualized using immunocytochemistry, and levels of the PAX6 transcription factor, which is expressed during neurogenesis, was assessed using RT-PCR. (TIF) [file pone.0019503.s005.tif]

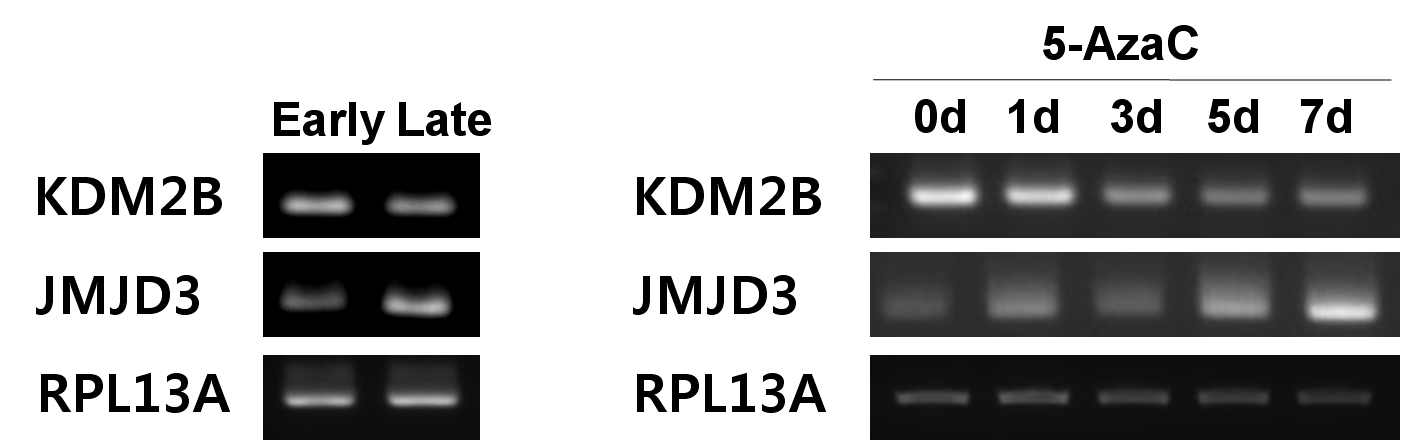

Supplement: Figure S6 — Expression levels of histone demethylases in early/late passages and 5-AzaC-treated hUCB-MSCs. RT-PCR analysis was performed to confirm the expression levels of histone demethylases, KDM2B and JMJD3. (TIF) [file pone.0019503.s006.tif]
