# Supplementary material for: DNA Methyltransferase Controls Stem Cell Aging by Regulating BMI1 and EZH2 through MicroRNAs
Source: PLoS One. 2011 May 10;6(5):e19503. doi: 10.1371/journal.pone.0019503 (PMC3091856; doi:10.1371/journal.pone.0019503)
Supplement: Methods S1 — Isolation and culture of hAD-MSCs. (DOCX) [file pone.0019503.s007.docx]

**Methods S1**

**Isolation and culture of hAD-MSCs**

Human adipose tissue-derived MSCs(hAD-MSCs) were isolated and cultured as previously described[[1](#_ENREF_1)]. In brief, two clones of hAD-MSCs were established from freshly excised mammary fat tissue from Ba-Ram plastic surgery hospital. Tissues were obtained from 20 to 30 year-old women during reduction mammoplasty. The hAD-MSCs were maintained in K-NAC medium supplemented with 2 mM N-acetyl-L-cysteine (Sigma-Aldrich, St. Louis, MO, USA) and L-ascorbic acid (0.2 mM, Sigma-Aldrich). For long term culture, cells were seeded at a density of 4x10^5^ cells/10cm-plate and subcultured cells when they reach 80~90% confluency.

1. Park JR, Jung JW, Lee YS, Kang KS (2008) The roles of Wnt antagonists Dkk1 and sFRP4 during adipogenesis of human adipose tissue-derived mesenchymal stem cells. Cell Prolif 41: 859-874.
