# Supplementary material for: DNA Methyltransferase Controls Stem Cell Aging by Regulating BMI1 and EZH2 through MicroRNAs
Source: PLoS One. 2011 May 10;6(5):e19503. doi: 10.1371/journal.pone.0019503 (PMC3091856; doi:10.1371/journal.pone.0019503)
Supplement: Table S1 — Primer sequences used for realtime qPCR (DOC) [file pone.0019503.s008.doc]

Table S1. Primer sequences used for realtime qPCR

| p16INK4A | F | 5'-GAAGGTCCCTCAGACATCCC-3' |
| --- | --- | --- |
|  | R | 5'-CCCTGTAGGACCTTCGGTGA-3' |
| p21WAF/CIP1 | F | ATTAGCAGCGGAACAAGGAG |
|  | R | R--CTGTGAAAGACACAGAACAG |
| EZH2 | F | 5'-GGGACAGTAAAAATGTGTCC-3' |
|  | R | 5'-TGCCAGCAATAGATGCTTTT-3' |
| BMI1 | F | 5'-TGCTGATGCTGCCAATGG-3' |
|  | R | 5'-TTACTTTCCGATCCAATCTG-3' |
| DNMT1^[1]^ | F | 5′-CGCTGTATCTAGCAAGGGTCA-3′ |
|  | R | 5′-TCGAATCTCGCGTAGTCTTG-3′ |
| DNMT3a^[1]^ | F | 5′-ACCACAGAGGCGGAAATACC-3′ |
|  | R | 5′-GTCTCCCTGCTGCTAACTGG-3′ |
| DNMT3b^[1]^ | F | 5′-CAGGAGACCTACCCTCCACA-3′ |
|  | R | 5′-TTACGTCGTGGCTCCAGTTA-3′ |
| COL-1 | F | 5′-GAGAGAGAGGCTTCCCTGGT-3′ |
|  | R | 5′-CACCACGATCACCACTCTTG-3′ |
| aP2 | F | 5′-TGCTTTTGTAGGTACCTGGA-3′ |
|  | R | 5′-CATAAACTCTCGTGGAAGTG-3′ |
| PAX6 | F | 5′-AACAGACACAGCCCTCACAAAC-3′ |
|  | R | 5′-CGGGAACTTGAACTGGAACTGAC-3′ |
| RPL13A | F | 5'-CATCGTGGCTAAACAGGTACTG-3' |
|  | R | 5'-GCACGACCTTGAGGGCAGCC-3' |

1. Deng T, Zhang Y (2009) Possible involvement of activation of P53/P21 and demethylation of RUNX 3 in the cytotoxicity against Lovo cells induced by 5-Aza-2'-deoxycytidine. Life Sci 84: 311-320.
