# Supplementary material for: DNA Methyltransferase Controls Stem Cell Aging by Regulating BMI1 and EZH2 through MicroRNAs
Source: PLoS One. 2011 May 10;6(5):e19503. doi: 10.1371/journal.pone.0019503 (PMC3091856; doi:10.1371/journal.pone.0019503)
Supplement: Table S2 — Promoter primer sequences used for ChIP analysis (DOC) [file pone.0019503.s009.doc]

Table S2. Promoter primer sequences used for ChIP analysis

| p16INK4A | F | 5’-CTCAAAGCGGATAATTCAAGAGC-3’ |
| --- | --- | --- |
|  | R | 5’-AAGCCTTAAGAACAGTGCCACAC-3’ |
| p21WAF/CIP1 | F | 5’-CGTGGTGGTGGTGAGCTAGA-3’ |
|  | R | 5’-CTGTCTGCACCTTCGCTCCT-3’ |
| miR-200c | F | 5’-GGCCTGAAGCTGCCTGACCC-3’ |
|  | R | 5’-GCAGCCAGCTAAGGGCTGGG-3’ |
| miR-214 | F | 5’-TGAAAAGGGCTGCTGTTTCCATGTA-3’ |
|  | R | 5’-GCCCACATGTAGTCAGAGTTCAGC-3’ |

1. Deng T, Zhang Y (2009) Possible involvement of activation of P53/P21 and demethylation of RUNX 3 in the cytotoxicity against Lovo cells induced by 5-Aza-2'-deoxycytidine. Life Sci 84: 311-320.
