# Supplementary material for: DNA Methyltransferase Controls Stem Cell Aging by Regulating BMI1 and EZH2 through MicroRNAs
Source: PLoS One. 2011 May 10;6(5):e19503. doi: 10.1371/journal.pone.0019503 (PMC3091856; doi:10.1371/journal.pone.0019503)
Supplement: Table S3 — Primers used for methyl specific PCR (DOCX) [file pone.0019503.s010.docx]

Table S3. Primers used for methyl specific PCR

| miR-200c p1 | M | F | 5’-GAGGGTTTTAAGTTGGTTAAGAAGC-3’ |
| --- | --- | --- | --- |
|  |  | R | 5’-AACGATAACGCAAACAATAACG-3’ |
|  | U | F | 5’-GGTTTTAAGTTGGTTAAGAAGTGG-3’ |
|  |  | R | 5’-AAACAATAACACAAACAATAACACA-3’ |
| miR-200c p2 | M | F | 5’-TTTATTAATTTTTTGCGTTTTTCGT-3’ |
|  |  | R | 5’-ATCTTACCCATATCGTTACAAACGT-3’ |
|  | U | F | 5’-TTTATTAATTTTTTGTGTTTTTTGT-3’ |
|  |  | R | 5’-CTTACCCATATCATTACAAACATT-3’ |
| miR-214 p1 | M | F | 5’-TCGAGTAGTTGGGATTATAGGTATTC-3’ |
|  |  | R | 5’-ACACTTTAAAAAACCGAAACGAA-3’ |
|  | U | F | 5’-TGAGTAGTTGGGATTATAGGTATTTGT-3’ |
|  |  | R | 5’-CCAACACTTTAAAAAACCAAAACA-3’ |
| miR-214 p2 | M | F | 5’-GAGATTAAGGCGGGTAGATTAC-3’ |
|  |  | R | 5’-CTAAATAACTAAAACTACAAACGCC-3’ |
|  | U | F | 5’-GAGATTAAGGTGGGTAGATTATGA-3’ |
|  |  | R | 5’-CCTAAATAACTAAAACTACAAACACC-3’ |
| p16^INK4A^ p1 | M | F | 5’-GAGATCGAGATTATTTCGGTTAAAAC-3’ |
|  |  | R | 5’-GCCCAAACTAAAATACAATAACGAA-3’ |
|  | U | F | 5’-ATTGAGATTATTTTGGTTAAAATGG-3’ |
|  |  | R | 5’-ACCCAAACTAAAATACAATAACAAA-3’ |
| p16^INK4A^ p2 | M | F | 5’-TGTAATTTTAATATTTTGGGAGGTC-3’ |
|  |  | R | 5’-CAACACCTTTTTAATAAAAACGAA-3’ |
|  | U | F | 5’-TAATTTTAATATTTTGGGAGGTTGA-3’ |
|  |  | R | 5’-ACAACACCTTTTTAATAAAAACAAA-3’ |
| p16^INK4A^ p3 | M | F | 5’-TTATTAGAGGGTGGGGCGGATCGC-3’ |
|  |  | R | 5’-GACCCCGAACCGCGACCG TAA-3’ |
|  | U | F | 5’-TTATTAGAGGGTGGGGTGGATTGT-3’ |
|  |  | R | 5’-CAACCCCAAACCACAACCATAA-3’ |
| p21^CIP1/WAF1^ p1 | M | F | 5’-TCGTTTAGGTTGGAGTGTAGTAGC-3’ |
|  |  | R | 5’-AAAAATTATCTAAACGTAATAACGAA-3’ |
|  | U | F | 5’-TGTTTAGGTTGGAGTGTAGTAGTGT-3’ |
|  |  | R | 5’-AAAAATTATCTAAACATAATAACAAA-3’ |
| p21^CIP1/WAF1^ p2 | M | F | 5’-TAAAATTAGTTAGGTATGGTGGCGT-3’ |
|  |  | R | 5’-ACTAAAATACAATAACGCGATCTCG-3’ |
|  | U | F | 5’-AAATATTAAAATTAGTTAGGTATGGTGGTG-3’ |
|  |  | R | 5’-AAATACAATAACACAATCTCAAC-3’ |
| p21^CIP1/WAF1^ p3 | M | F | 5’-TTGGGCGCGGATTCGTC-3’ |
|  |  | R | 5’-CTAAACCGCCGACCCGA-3’ |
|  | U | F | 5’-TTAGTTTTTTGTGGAGTTG-3’ |
|  |  | R | 5’-CTCAACTCTAAACCACCAA-3’ |
